# Mortality prediction models in acute respiratory failure treated with extracorporeal membrane oxygenation: it must be firstly designed for clinicians and beside use

**DOI:** 10.1186/cc13967

**Published:** 2014-07-03

**Authors:** Matthieu Schmidt, David Pilcher

**Affiliations:** 1 Medical-Surgical Intensive Care Unit, iCAN, Institute of Cardiometabolism and Nutrition, Hôpital de la Pitié-Salpêtrière, Assistance Publique-Hôpitaux de Paris, 47-89 boulevard de l’Hopital, Paris 75013, France; 2 Australian and New Zealand Intensive Care Research Centre, Department of Epidemiology and Preventive Medicine, School of Public Health, Monash University, Commercial Rd, Melbourne, Vic 3004, Australia

## 

In a recent issue of *Critical Care*, we read with great interest the article by Enger and colleagues [1] regarding the development of novel mortality prediction models on extracorporeal membrane oxygenation (ECMO) in acute respiratory failure and the comparison of its performance with the ECMOnet (Extracorporeal Membrane Oxygenation Network) [2], PRESERVE (Predicting Death for Severe Acute Respiratory Distress Syndrome on Veno-venous Extracorporeal Membrane Oxygenation) [3], and Sequential Organ Failure Assessment scores. We commend the efforts made by the authors to externally validate the ECMOnet and the PRESERVE scores, but we recognize that a problem common to all prediction models, including their own, is that inevitably performance is better in the dataset from which they were derived. A potential limitation of the approach taken by Enger and colleagues is that the calculation of predicted mortality requires the use of a computer-generated algorithm which may limit immediate ease of use and applicability by clinicians. Prediction scores (such as the ECMOnet and the PRESERVE) which contain only categorical variables may be better for beside use. Derivation of a score from the pre-ECMO model and the model day 1 (which contained 4 out of 5 and 7 out of 8 continuous predictors) in the study by Enger and colleagues would have further decreased the discrimination of both models [4]. The recently published Respiratory Extracorporeal Membrane Oxygenation Survival Prediction (RESP) score, derived from over 2,000 patients from the Extracorporeal Life Support Organization (ELSO) registry [5,6], may provide another mortality prediction tool which could also be assessed.

Lastly, peak inspiratory pressure (PIP) was used as a substitute for plateau pressure in the validation of the PRESERVE score. It would have been helpful of the authors to provide the PIP threshold used. Substitution of the same threshold for PIP as used for the plateau pressure (that is, 30 cmH_2_O) would have led to an overestimated low pre-ECMO compliance and potentially impaired performance of the PRESERVE score in this cohort.

## Authors’ response

Tone Bull Enger, Alois Philipp, Vibeke Videm and Thomas Müller

We thank Schmidt and Pilcher for their comments on our study [1] and agree that external validation of predictive scores usually shows varying degrees of worse performance than internal validation. However, we have not been able to find suitable data from a sufficiently big cohort for external validation of our score. It seems that few centers systematically register results from blood tests such as lactate, hemoglobin, or fibrinogen, which were found to improve prediction in our population. We therefore encourage registration of such variables, as the usefulness of a score depends on the contents of the database it is designed from.

We suggest that the practice of categorizing continuous variables for easier use of prediction scores be discouraged, as it decreases performance and is unnecessary nowadays. Instead, using online calculators that can also be available in bedside mobile versions is a better approach. We are in the process of making a publically available online version of our score.

The RESP score from the ELSO registry [6], which does not include laboratory data either, was not published when our manuscript was accepted. It will be very interesting to validate this score in our population and compare it with the performance of our score.

Because most hospitals in Germany use pressure-controlled ventilation in severe acute respiratory distress syndrome, plateau pressure is not available in our database. To evaluate the applicability of the PRESERVE score [3] for our study cohort, PIP was used as a substitute for plateau pressure using the same threshold (30 cmH_2_O). We agree that the use of PIP instead may result in a slight overestimation of impaired lung compliance.

## Abbreviations

ECMO: Extracorporeal membrane oxygenation; ECMOnet: Extracorporeal Membrane Oxygenation Network; ELSO: Extracorporeal Life Support Organization; PIP: Peak inspiratory pressure; PRESERVE: Predicting Death for Severe Acute Respiratory Distress Syndrome on Veno-venous Extracorporeal Membrane Oxygenation; RESP: Respiratory Extracorporeal Membrane Oxygenation Survival Prediction.

## Competing interests

MS and DP declare that they have no competing interests.
